# Analysis of MADS-box genes revealed modified flowering gene network and diurnal expression in pineapple

**DOI:** 10.1186/s12864-019-6421-7

**Published:** 2020-01-02

**Authors:** Xiaodan Zhang, Mahpara Fatima, Ping Zhou, Qing Ma, Ray Ming

**Affiliations:** 1 Center for Genomics and Biotechnology, Fujian Provincial Key Laboratory of Haixia Applied Plant Systems Biology, Fujian Agriculture and Forestry University, Fuzhou, 350002 Fujian China; 2 Department of Plant Biology, University of Illinois at Urbana-Champaign, Urbana, IL 61801 USA; 3 Fujian Academy of Agricultural Sciences, Fruit Research Institute, Fuzhou, 350013 Fujian China

**Keywords:** CAM photosynthesis, Diurnal clock, MADS-box genes, Pineapple

## Abstract

**Background:**

Pineapple is the most important crop with CAM photosynthesis, but its molecular biology is underexplored. MADS-box genes are crucial transcription factors involving in plant development and several biological processes. However, there is no systematic analysis of MADS-box family genes in pineapple (*Ananas comosus*).

**Results:**

Forty-eight MADS-box genes were identified in the pineapple genome. Based on the phylogenetic studies, pineapple MADS-box genes can be divided into type I and type II MADS-box genes. Thirty-four pineapple genes were classified as type II MADS-box genes including 32 MIKC-type and 2 Mδ-type, while 14 type I MADS-box genes were further divided into Mα, Mβ and Mγ subgroups. A majority of pineapple MADS-box genes were randomly distributed across 19 chromosomes. RNA-seq expression patterns of MADS-box genes in four different tissues revealed that more genes were highly expressed in flowers, which was confirmed by our quantitative RT-PCR results. There is no FLC and CO orthologs in pineapple. The loss of FLC and CO orthologs in pineapple indicated that modified flowering genes network in this tropical plant compared with *Arabidopsis*. The expression patterns of MADS-box genes in photosynthetic and non-photosynthetic leaf tissues indicated the potential roles of some MADS-box genes in pineapple CAM photosynthesis. The 23% of pineapple MADS-box genes showed diurnal rhythm, indicating that these MADS-box genes are regulated by circadian clock.

**Conclusions:**

MADS-box genes identified in pineapple are closely related to flowering development. Some MADS-box genes are involved in CAM photosynthesis and regulated by the circadian clock. These findings will facilitate research on the development of unusual spiral inflorescences on pineapple fruit and CAM photosynthesis.

## Background

MADS-box genes play a crucial role in plant development, especially in flower development. The term ‘MADS’ was derived from four members of the MADS family in fungi, plants and animals: MCM1 in yeast, AGAMOUS in *Arabidopsis*, DEFICIENS in snapdragon, and SERUM RESPONSE FACTOR in human [1–5]. MADS-box genes possess a highly conserved MADS domain that consists of roughly 60 amino acids at the amino-terminal end of the protein, followed by the I domain, the K domain and the C region from N-termini to C-termini [6, 7]. K domain is also highly conserved, while I domain and C region are quite variable. MADS domain encodes a DNA binding and dimerization function, and K domain encodes a coiled-coil motif that could possibly serve the function of mediating protein-protein interaction [1, 8].

Because of the similarities between the DNA-binding domains of MADS-box genes and subunit A of topoisomerase IIA (TOPOIIA-A), it was postulated that one copy of TOPOIIA-A was the progenitor MADS-box transcription factor [9]. In the second duplication, recent common ancestor was divided into two MADS-box types: type I (SRF-like) and type II (MEF2-like) [9, 10]. type I MADS-box genes can be further classified into Mα, Mβ and Mγ, while Type II s can be divided into MIKC-type and Mδ-type [11]. To date, MADS-box genes have been identified and classified in many dicot and monocot plants including *Arabidopsis* [12], *Vitis vinifera* [13], cucumber [14], banana [15], *Brachypodium* [16], wheat [17], soybean [18] and Chinese jujube [19]. The first group of MADS genes to be characterized in plant were floral organ identity genes, or ABC genes [20]. Floral organ identity genes can be summarized in the ABC model and later expanded to the ABCDE models [21]. In *Arabidopsis*, 107 MADS-box genes have been identified and their functions have also been determined [12, 22].

Pineapple (*Ananas comosus* (L.) Merr.) is an economically valuable fruit crop cultivated in tropical regions. But the molecular and genetic mechanisms of flower and fruit development have not been explored extensively. MADS-box family genes were reportedly playing an important role in the flower and fruit development process [22]. Analyzing the MADS-box genes in pineapple will be able to facilitate studies of molecular mechanisms in pineapple flower and fruit development and further characterize the function of MADS-box genes in pineapple. Meanwhile, pineapple is a fruit crop utilizing Crassulacean Acid Metabolism (CAM), which is an efficient CO_2_ fixation pathway [23]. Understanding the circadian rhythm of pineapple MADS-box genes can provide a foundation for elucidating CAM and CAM-related application in crop improvement.

In this study, the MADS-box genes in pineapple were identified and then classified based on their phylogenetic relationships. Gene structures and conserved motifs of pineapple MADS-box genes were analyzed, and the chromosome locations were mapped. The tissue-specific and diurnal expression patterns of MADS-box genes were evaluated. The results can improve our understanding for the evolution and functions of MADS-box genes in pineapple.

## Results

### Identification and classification of MADS-box genes in pineapple

Initially, 44 pineapple MADS-box genes were identified by Hidden Markov Model (HMM) search. To carry out an exhaustive search for MADS-box genes, BLASTP was conducted to search the pineapple genome database using MADS-box protein sequences in *Arabidopsis* and rice as queries. Finally, a total of 48 MADS-box genes were identified in the pineapple genome (Table 1) and further confirmed by NCBI Conserved Domain Database. The CDS length of pineapple MADS-box genes ranged from 180 bp (Aco030553.1) to 4569 bp (Aco027629.1). The relative molecular mass varied from 6.68 kDa to 166.54 kDa, and protein IP ranged from 4.80 to 11.23.

**Table 1:** MADS-box gene family identified in pineapple

Gene ID	Gene name	Type	Chr.	Length of CDS (bp)	# of Exons	# of Introns	IP	MW (kDa)
*AcMADS1*	Aco001069.1	MIKC	Chr2	882	9	8	9.48	33.68
*AcMADS2*	Aco002729.1	MIKC	Chr6	897	9	8	9.22	33.32
*AcMADS3*	Aco003018.1	MIKC	Chr6	744	8	7	7.05	28.01
*AcMADS4*	Aco003667.1	MIKC	Chr17	803	11	10	7.63	30.19
*AcMADS5*	Aco004028.1	MIKC	Chr15	693	8	7	8.44	25.96
*AcMADS6*	Aco004785.1	MIKC	Chr5	717	7	6	9.23	27.27
*AcMADS7*	Aco004839.1	MIKC	Chr7	747	8	7	9.18	28.43
*AcMADS8*	Aco004987.1	Mα	Chr7	672	1	0	9.26	24.16
*AcMADS9*	Aco004988.1	Mα	Chr7	1311	1	0	7.66	45.32
*AcMADS10*	Aco006017.1	MIKC	Chr16	591	6	5	7.78	22.88
*AcMADS11*	Aco007995.1	MIKC	Chr21	552	8	7	5.80	20.77
*AcMADS12*	Aco007999.1	MIKC	Chr21	702	7	6	9.49	26.89
*AcMADS13*	Aco008359.1	MIKC	Chr19	720	6	5	7.16	27.54
*AcMADS14*	Aco008435.1	Mα	Chr19	996	1	0	6.93	35.79
*AcMADS15*	Aco008623.1	Mα	Chr9	687	1	0	6.86	24.66
*AcMADS16*	Aco009993.1	MIKC	Chr10	762	7	6	9.30	29.60
*AcMADS17*	Aco011341.1	MIKC	Chr1	705	7	6	9.35	26.96
*AcMADS18*	Aco011374.1	Mβ	Chr1	1263	2	1	9.87	49.21
*AcMADS19*	Aco011677.1	Mα	Chr8	573	1	0	6.35	21.25
*AcMADS20*	Aco012428.1	MIKC	Chr1	1074	9	8	8.19	39.44
*AcMADS21*	Aco013229.1	MIKC	Chr24	639	7	6	9.11	24.13
*AcMADS22*	Aco013324.1	Mα	Chr15	717	1	0	8.68	25.85
*AcMADS23*	Aco013644.1	Mγ	Chr13	762	1	0	9.39	28.25
*AcMADS24*	Aco013736.1	Mδ	Chr13	1053	10	9	5.61	39.54
*AcMADS25*	Aco014671.1	MIKC	Chr20	711	7	6	8.43	26.95
*AcMADS26*	Aco015104.1	MIKC	Chr1	759	6	5	9.04	27.92
*AcMADS27*	Aco015105.1	MIKC	Chr1	741	8	7	8.43	28.22
*AcMADS28*	Aco015487.1	MIKC	Chr3	726	8	7	9.26	27.53
*AcMADS29*	Aco015491.1	MIKC	Chr3	762	8	7	9.59	28.31
*AcMADS30*	Aco015492.1	MIKC	Chr3	492	3	2	11.23	18.66
*AcMADS31*	Aco016643.1	MIKC	Chr8	627	7	6	9.12	24.01
*AcMADS32*	Aco017499.1	MIKC	Chr22	605	6	5	8.88	23.37
*AcMADS33*	Aco017563.1	MIKC	Chr9	744	8	7	8.67	28.16
*AcMADS34*	Aco017589.1	MIKC	Chr9	768	5	4	9.56	18.24
*AcMADS35*	Aco018015.1	MIKC	Chr1	645	6	5	6.37	24.37
*AcMADS36*	Aco019026.1	Mδ	Chr2	288	3	2	8.71	10.84
*AcMADS37*	Aco019039.1	Mα	Chr20	630	5	4	9.64	23.14
*AcMADS38*	Aco019365.1	MIKC	Chr5	594	7	6	9.28	22.88
*AcMADS39*	Aco019839.1	Mγ	Chr15	360	5	4	9.01	13.99
*AcMADS40*	Aco019842.1	MIKC	Chr15	420	5	4	9.98	15.13
*AcMADS41*	Aco022101.1	Mγ	Chr4	804	1	0	8.37	29.60
*AcMADS42*	Aco025594.1	MIKC	scaffold_679	468	5	4	9.56	18.24
*AcMADS43*	Aco027629.1	Mγ	scaffold_622	4569	19	18	4.80	166.54
*AcMADS44*	Aco027879.1	MIKC	scaffold_1163	693	8	7	8.77	25.90
*AcMADS45*	Aco028086.1	Mβ	scaffold_1517	1056	3	2	5.00	39.81
*AcMADS46*	Aco030142.1	MIKC	Chr22	525	5	4	6.85	19.56
*AcMADS47*	Aco030553.1	MIKC	scaffold_1319	180	1	0	10.60	6.68
*AcMADS48*	Aco030656.1	Mα	scaffold_2479	315	5	4	6.11	11.94

**Fig. 1:**
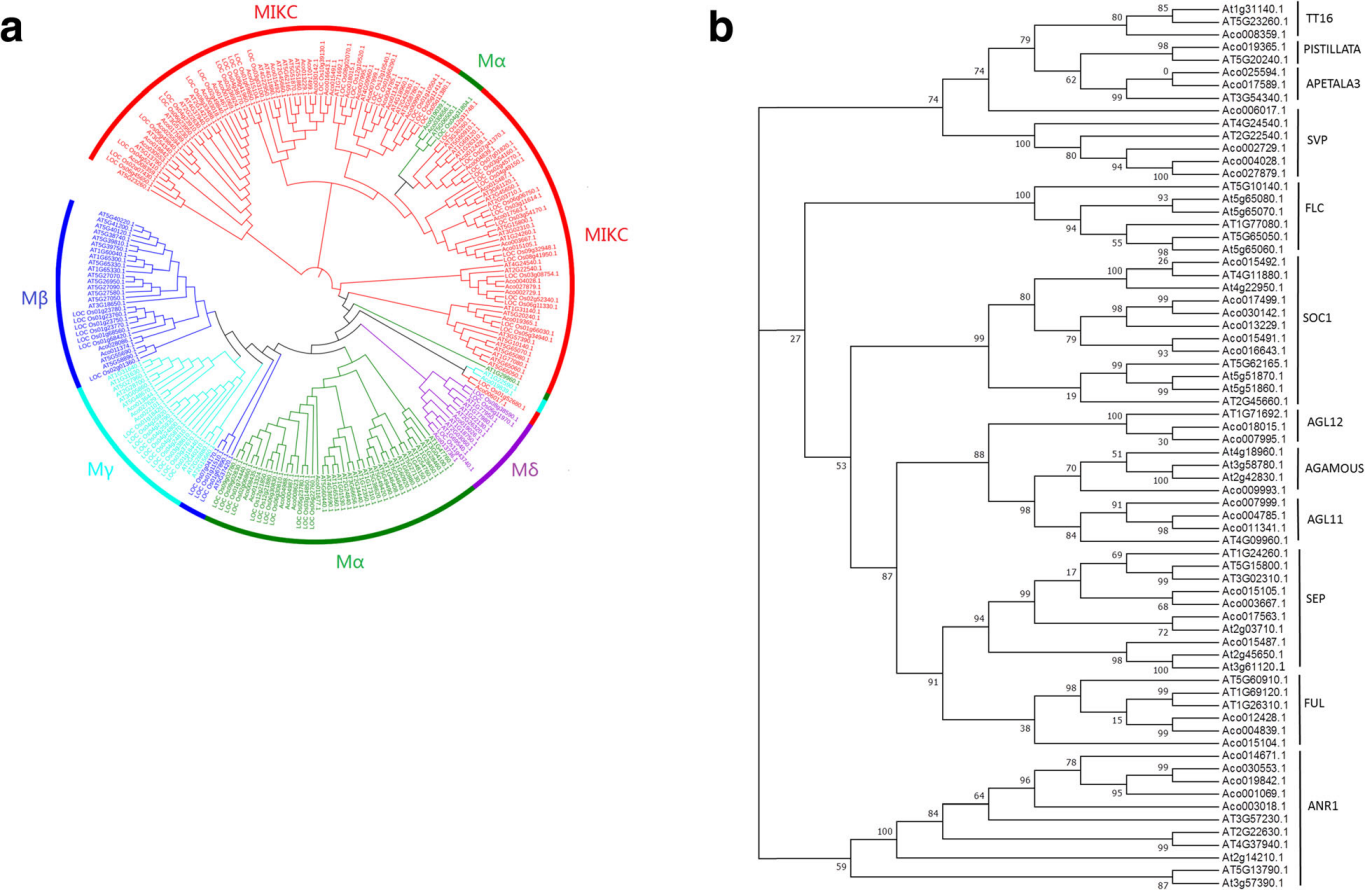
**a** Phylogenetic analysis of the MADS-box genes from Arabidopsis, rice and pineapple. **b** Phylogenetic analysis of the type II MADS-box genes from *Arabidopsis* and pineapple

In order to study the evolutionary relationship between pineapple MADS-box genes and the known MADS-box genes from *Arabidopsis* and rice, multiple sequence alignments were conducted and then a phylogenetic tree was constructed based on amino acids of MADS-box genes in pineapple, *Arabidopsis* and rice. Thirty-four pineapple genes were classified as type II MADS-box genes including 32 MIKC-type and 2 Mδ-type (Fig. 1a). Fourteen type I MADS-box genes were further divided into Mα, Mβ and Mγ subgroups. Mα was the type I subgroup with the most genes. Eight out of 14 type I genes were classified as Mα subgroup, while 2 and 4 type I genes were classified into Mβ and Mγ subgroup, respectively (Fig. 1a). 32 MIKC-type pineapple genes were further divided into 11 clusters: TT16, APETALA3, PISTILLATA, SVP, ANR1, SEP, FUL, AGL12, AGAMOUS, AGL11 and SOC1 (Fig. 1b).

### Gene structure and conserved motif analysis

**Fig. 2:**
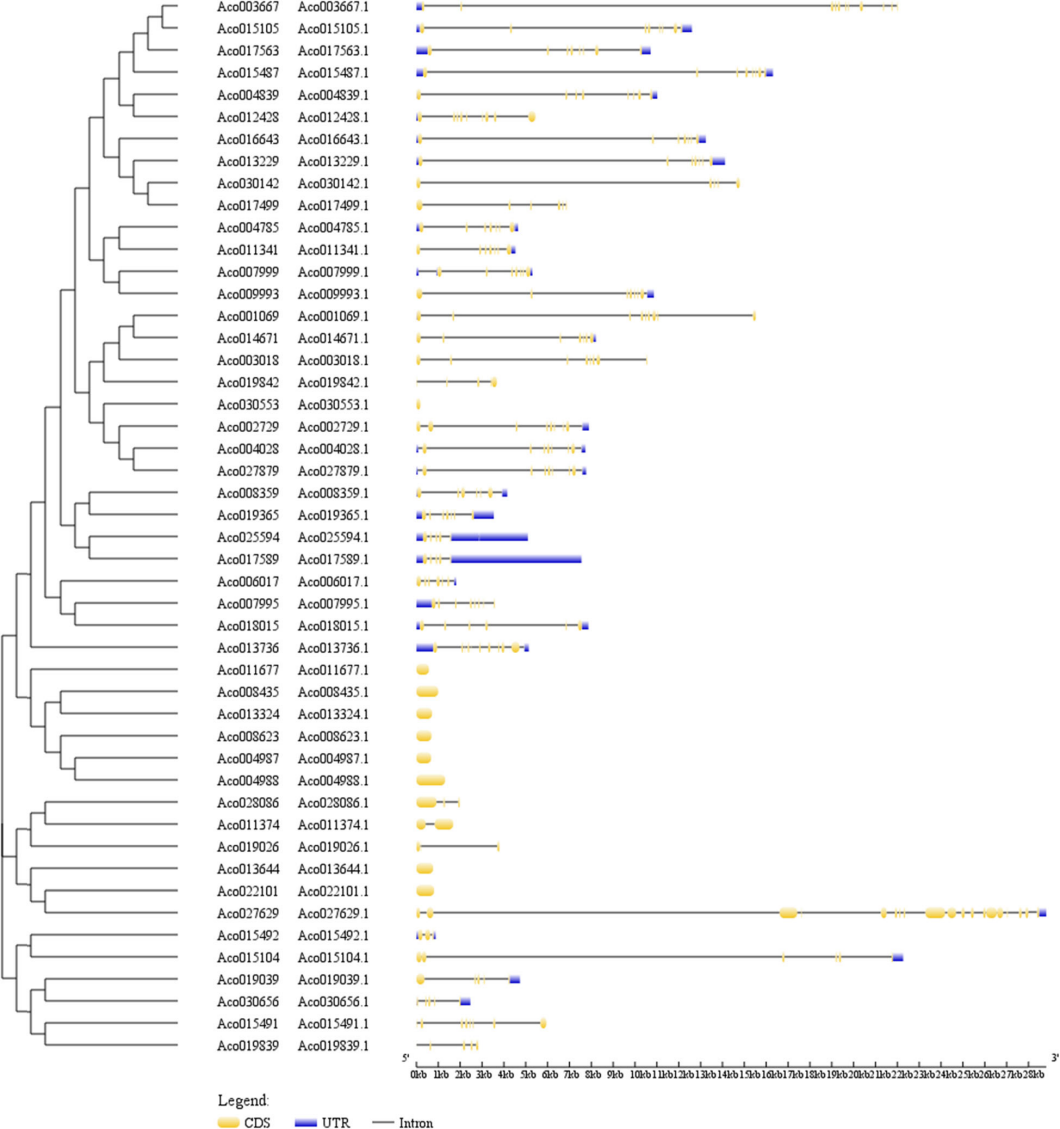
Phylogenetic relationship and gene structure analysis of MADS in pineapple

To explore the structural evolution of MADS-box genes in pineapple, structural arrangements of MADS-box genes were examined by Gene Structure Display Server. The result showed that the closely related genes were usually more similar in gene structure, such as genes *Aco004785.1*, *Aco011341.1*, *Aco007999.1* and *Aco009993.1*, which all had 7 exons. However, some closely related genes showed significant difference in structural arrangements (Fig. 2). For instance, *Aco022101.1* possesses only one exon, while *Aco027629.1*, its closely related gene, had 19 exons. Furthermore, pineapple MADS-box genes contained exons ranging from 1 to 19. Nine out of 48 MADS-box genes had only one exon, and those genes with one exon except for *Aco030553.1* belong to type I. The exon number of most pineapple MADS-box genes was less than 10, only three genes *Aco013736.1*, *Aco003667.1* and *Aco027629.1* had 10, 11 and 19 exons, respectively (Fig. 2).

**Fig. 3:**
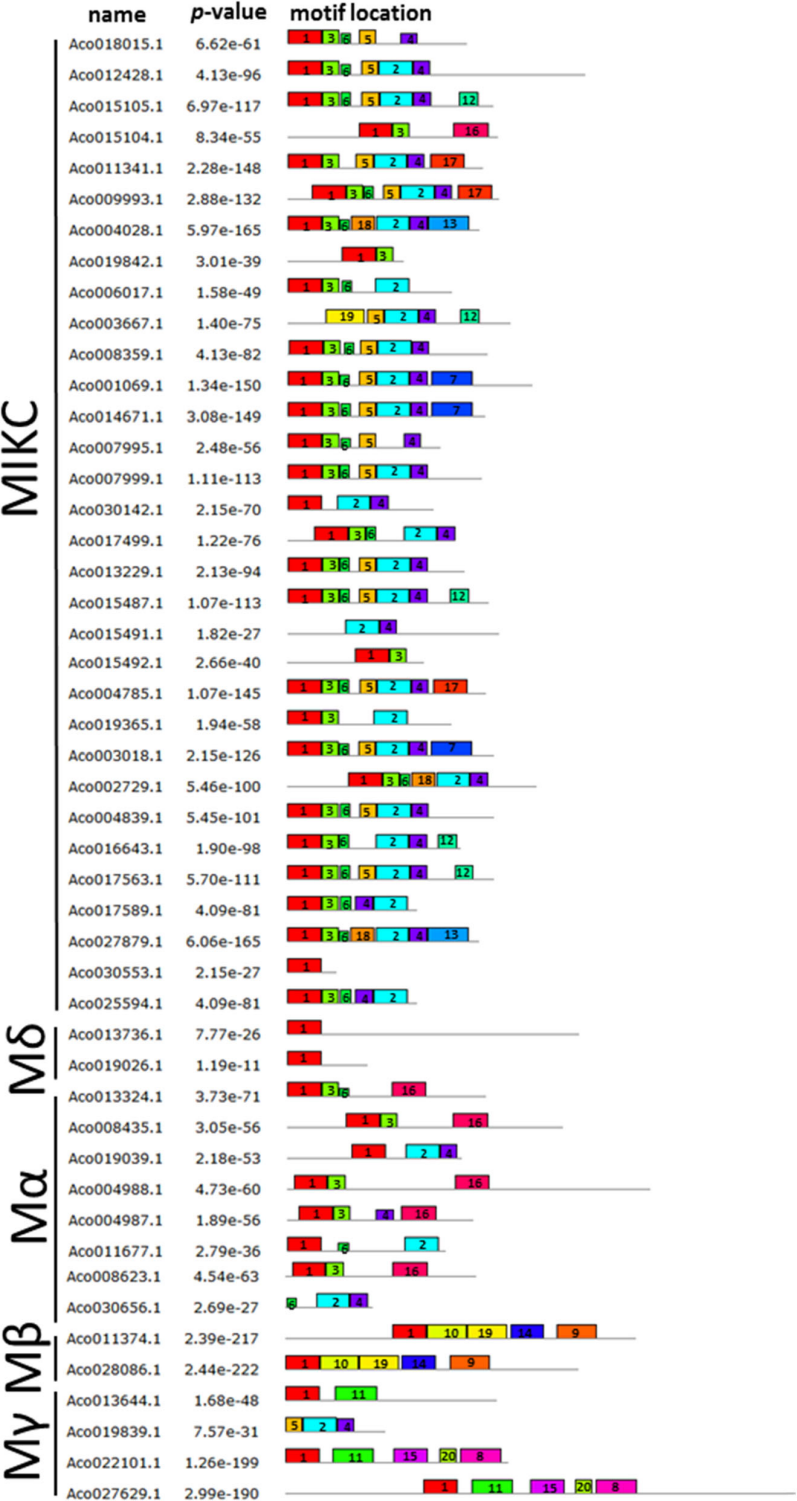
Conserved motif analysis of pineapple MADS-box genes

MEME software was used to analyze motifs in the MADS-box proteins. Twenty conserved motifs were identified (Fig. 3) and these conserved motifs were annotated by SMART program. Motif 1, 3, 7 and 11 are MADS domains, motif 2 represents K domain, and motif 6 is C domain. All of MADS-box genes (except for 4 genes: *Aco003667.1, Aco015492.1, Aco030656.1* and *Aco019839.1*) contained motif 1, and the 4 genes without motif 1 all contained motif 2. Meanwhile, motif 2 was identified in the majority of type II MADS-box genes, while it was only discovered in four type I genes (*Aco019039.1*, *Aco011677.1*, *Aco030656.1* and *Aco019839.1*). Genes in the same group tend to have commonly shared motifs. For example, Mδ-type group includes *Aco013736.1* and *Aco019026.1* contained only motif 1. *Aco022101.1* and *Aco027629.1*, in Mγ group, both possessed motifs 1, 8, 11, 15 and 20.

### Location on chromosomes of pineapple MADS-box genes

**Fig. 4:**
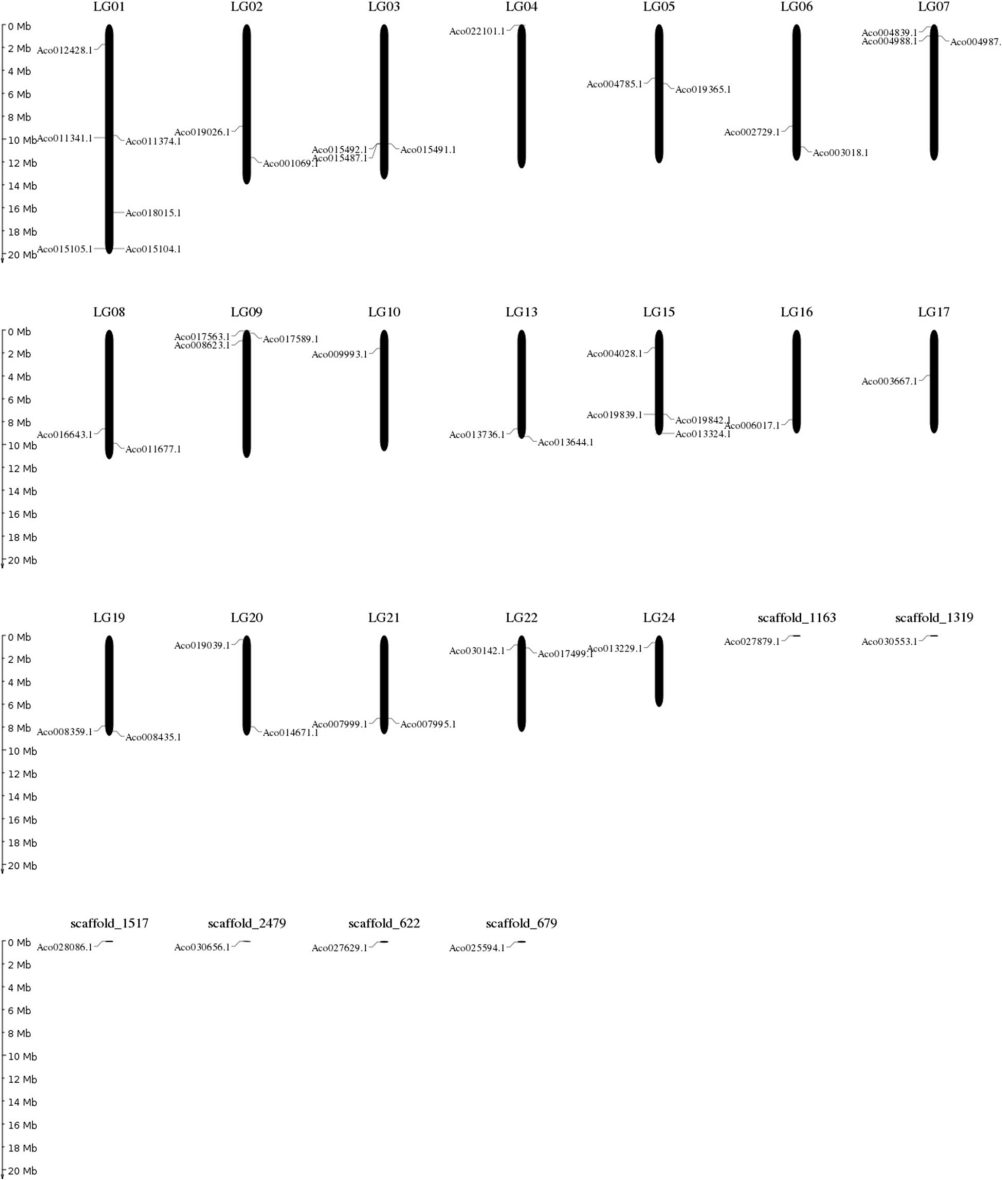
Distribution of MADS-box genes in pineapple linkage groups (LGs)

The majority of pineapple MADS-box genes (42 out of 48) were randomly distributed across 19 chromosomes, while only 6 genes were scattered in 6 scaffolds that could not be assigned to chromosomes (Table 1, Fig. 4). Six genes (12.5%) were on chromosome 1, followed by 4 genes (8.3%) on chromosome 15. Type II MADS-box genes were mapped to 18 chromosomes (except from chromosome 4), while type I MADS-box genes were scattered to only 9 chromosomes due to fewer members. Out of type I genes, Mα group genes were distributed on chromosomes 7, 8, 9, 15, 19 and 20, whereas two Mβ group genes were clustered across chromosomes 1 and scafford_1517. Genes in Mγ group were located on chromosomes 4, 13 and 15.

### Expression analysis of the pineapple MADS-box genes in different tissues

**Fig. 5:**
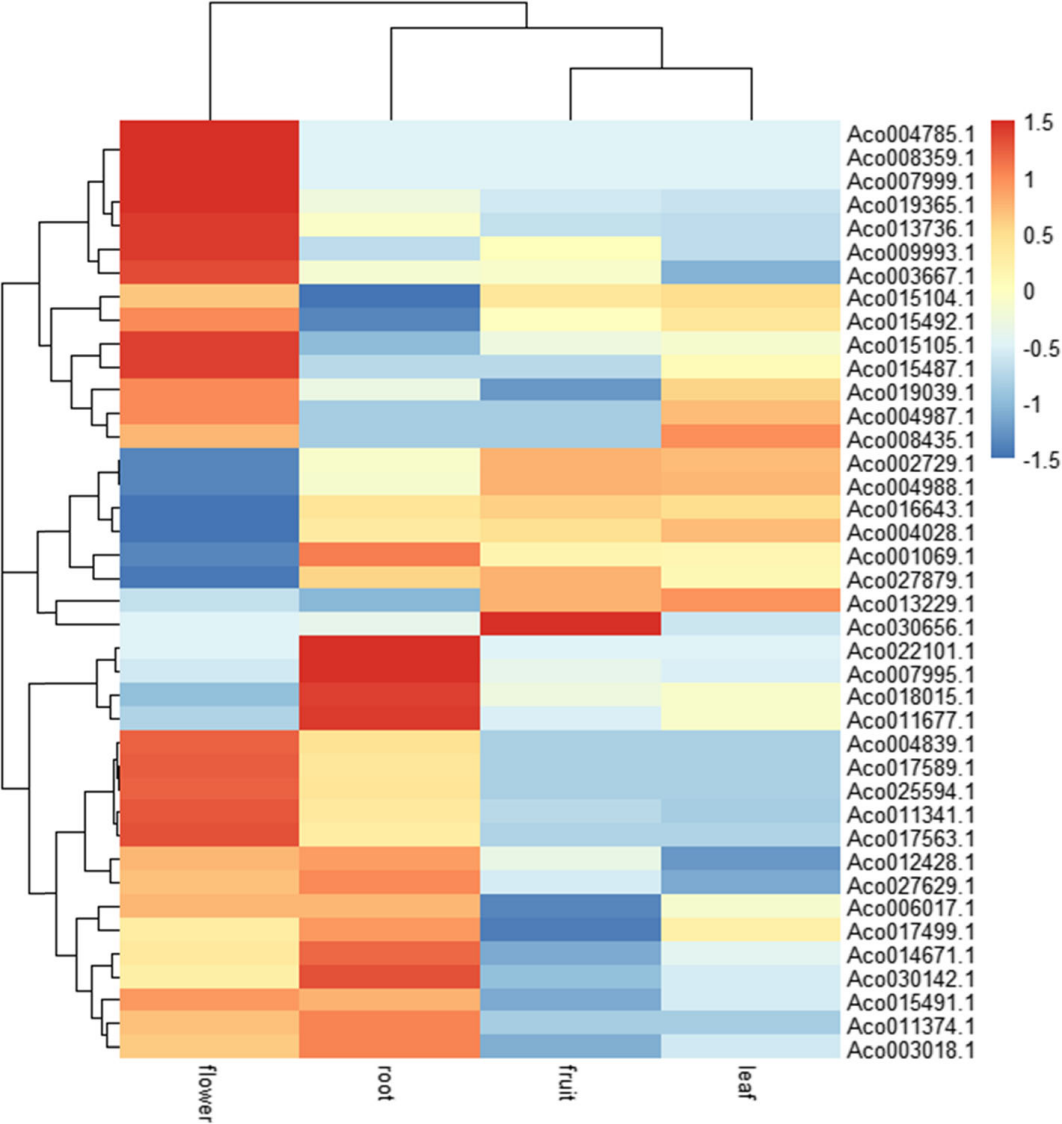
A heat map of tissue-specific expression data of MADS-box genes in pineapple

To investigate the expression patterns of pineapple MADS-box genes in different tissues, RNA-seq libraries prepared from four pineapple tissues: leaf, flower, root and fruit were constructed and RNA-seq analysis was further performed to obtain FPKM values of MADS-box genes in pineapple. Forty MADS-box genes were expressed in at least one tissue, while the other 8 genes (*Aco019026.1*, *Aco008623.1*, *Aco013644.1*, *Aco019842.1*, *Aco019839.1*, *Aco013324.1*, *Aco030553.1* and *Aco028086.1*) were not detectable in any of those four tissues. Therefore, 8 genes with no detectable expression (FPKM value equals “0” in all four tissues) were filtered out and the expression level of 40 genes was shown in a heat map (Fig. 5).

RNA-seq expression profile of pineapple MADS-box genes revealed that a majority of genes were highly expressed in flower. Besides, some genes, such as *Aco019365.1*, *Aco017589.1* and *Aco025594.1*, were expressed much higher in flower than in other tissues. In leaf tissues, many genes had relatively lower expression, but some genes (*Aco027629.1* and *Aco002729.1*) expressed higher in leaves than in flowers. In fruit tissue, a few genes, such as *Aco002729.1*, *Aco016643.1* and *Aco013229.1* showed high expression level. Two genes, *Aco007995.1* and *Aco018015.1*, were highly expressed in root, and *Aco022101.1* was only expressed in root.

**Fig. 6:**
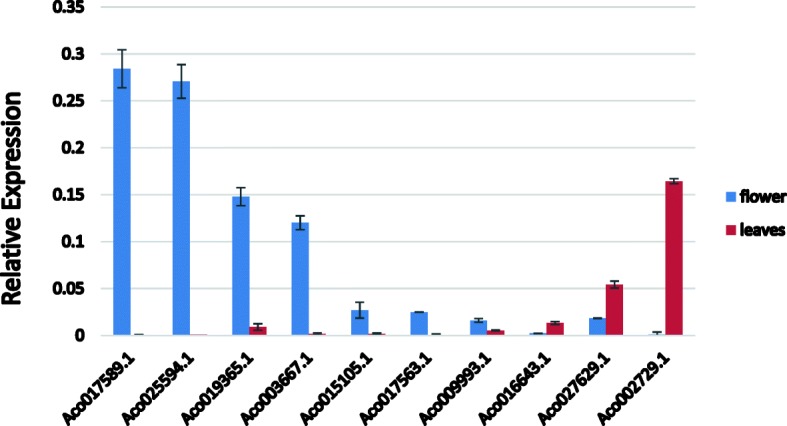
Relative expression of MADS-box genes in pineapple flower and leaves by qRT-PCR

Ten MADS-box genes were randomly selected for quantitative RT-PCR analysis in flower and leaf tissues to verify the RNA-seq data (Fig. 6). The qRT-PCR results confirmed that most of MADS-box genes had high expression in flower and had low expression in leaves. However, a few genes, such as *Aco027629.1* and *Aco002729.*1, expressed higher in leaves, which exhibited the same trend as RNA-seq data. These results showed that our RNA-seq data is suitable for investigating the expression patterns of MADS genes in different tissues of pineapple.

### Expression analysis of pineapple MADS-box genes in green tip and white base leaves

**Fig. 7:**
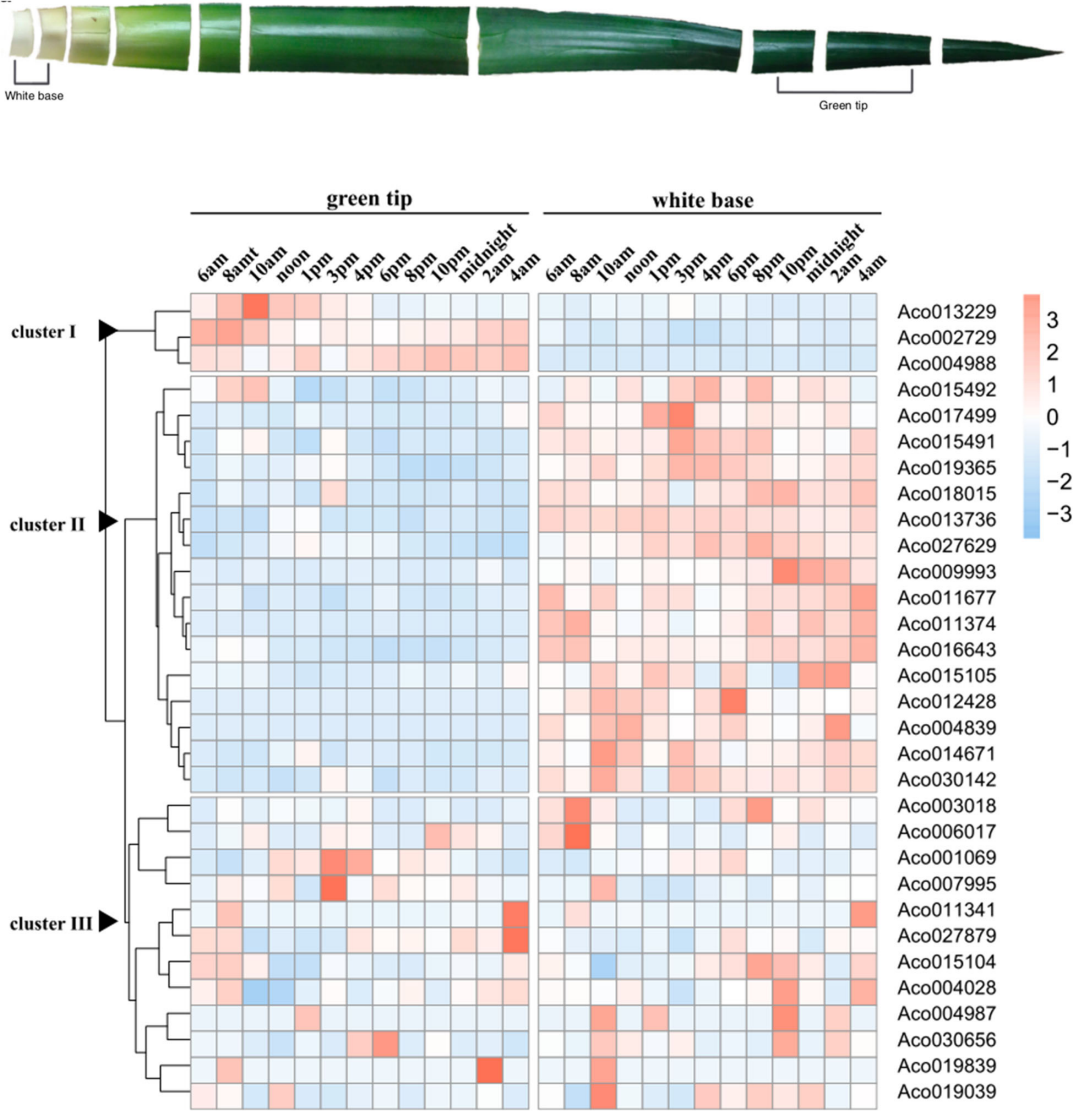
Expression profiles of pineapple MADS-box genes in both photosynthetic and non-photosynthetic leaf tissues

Pineapple is a CAM plant that achieves greater net CO_2_ uptake than their C_3_ and C_4_ counterparts [24]. To investigate the potential roles of MADS-box genes in pineapple CAM photosynthesis, we studied the expression pattern of MADS-box genes in photosynthetic (green tip) and non-photosynthetic (white base) leaf tissues. The green and white leaves are physiologically different, the green tip has very high concentration of chlorophyll, while white base contains extremely low chlorophyll concentration, which shows the difference of green and white leaves in photosynthetic rate [25]. The genes with no detectable expression and low expression (FPKM less than 1 in both tissues) were filtered out. As shown in Fig. 7, MADS-box genes can be classified into three clusters. Over the 24-h period, the expression level of cluster I genes in green tip leaf was higher than that in white base leaf. However, the cluster II genes showed opposite expression: genes in white base expressed higher than in green tip leaf. In cluster III, genes did not exhibit obvious differential expression between green tip and white base tissues. Meanwhile, some MADS-box genes showed peak expression at certain time period in either green tip or white base. For example, *Aco012428.1* had highest expression at 6 pm in white base leaf, while *Aco027629.1* exhibited highest expression at 12 am in green tip leaf.

**Fig. 8:**
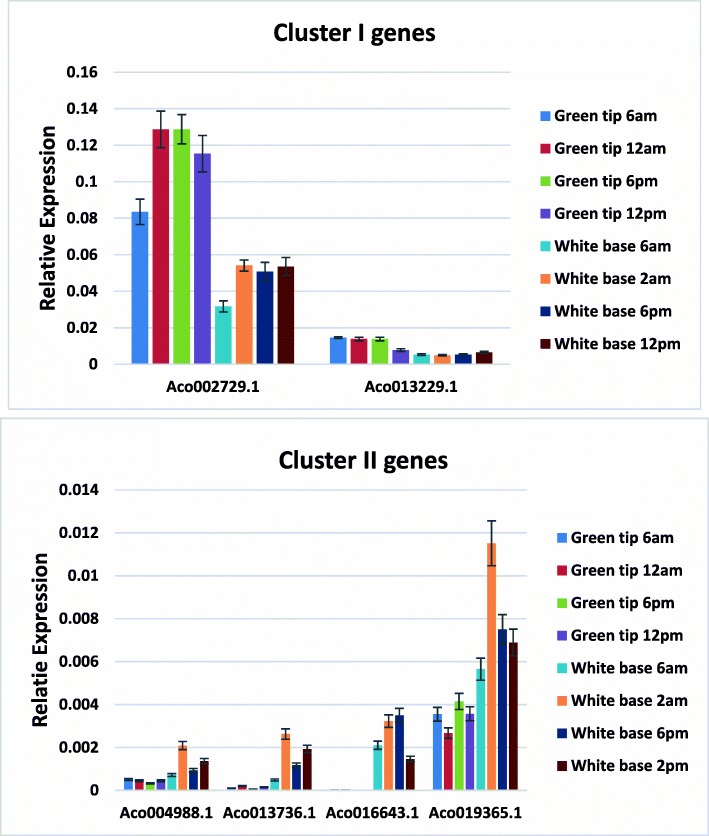
Relative expression of cluster l and ll MADS-box genes in green and white leaves at different time points by qRT-PCR

There are 14 genes in cluster I and II, we chose 6 genes for qRT-PCR analysis to verify their expression level in green and white leaves (Fig. 8). According to qRT-PCR results, the genes in cluster I also showed the similar expression pattern: expressed higher in green tip leaves than white base leaves, and cluster II genes had higher expression in white base leaves. Besides, our qRT-PCR results confirmed that *Aco027629.1* had highest expression at 12 am in green tip leaves.

### Diurnal expression analysis of pineapple MADS-box genes

To identify the circadian expression pattern of MADS-box genes in pineapple, RNA-Seq data of pineapple green tip and white base leaf tissues over 24-h period were used to determine MADS-box genes whose expression patterns fit the model of cycling genes in Haystack [26]. Transcription factors with a strong correlation (*r* > 0.7) were empirically considered as genes with diurnal rhythm [27], we used the same correlation cutoff as the threshold for analyzing diurnal expression pattern of MADS-box genes. 11 out of 48 (23%) of MADS-box genes were cycling in either green tip or white base leaf tissues. Out of these cycling genes, 4 genes (*Aco013229.1*, *Aco015104.1*, *Aco004028.1* and *Aco019365.1*), which all belong to type II group, were cycling in both green tip and white base leaf tissues (Table 2).

**Table 2:** Diurnal expression pattern of pineapple MADS-box genes

pineapple gene	types	gropus	description
Aco004988.1	type I	Mα	Cycling in Green tip leaf tissue
Aco015492.1	type II	MIKC	Cycling in Green tip leaf tissue
Aco015492.1	type II	MIKC	Cycling in Green tip leaf tissue
Aco016643.1	type II	MIKC	Cycling in Green tip leaf tissue
Aco011677.1	type I	Mα	Cycling in White base leaf tissue
Aco012428.1	type II	MIKC	Cycling in White base leaf tissue
Aco018015.1	type II	MIKC	Cycling in White base leaf tissue
Aco013229.1	type II	MIKC	Cycling in both green tip and white base leaf tissue
Aco004028.1	type II	MIKC	Cycling in both green tip and white base leaf tissue
Aco015104.1	type II	MIKC	Cycling in both green tip and white base leaf tissue
Aco019365.1	type II	MIKC	Cycling in both green tip and white base leaf tissue

**Fig. 9:**
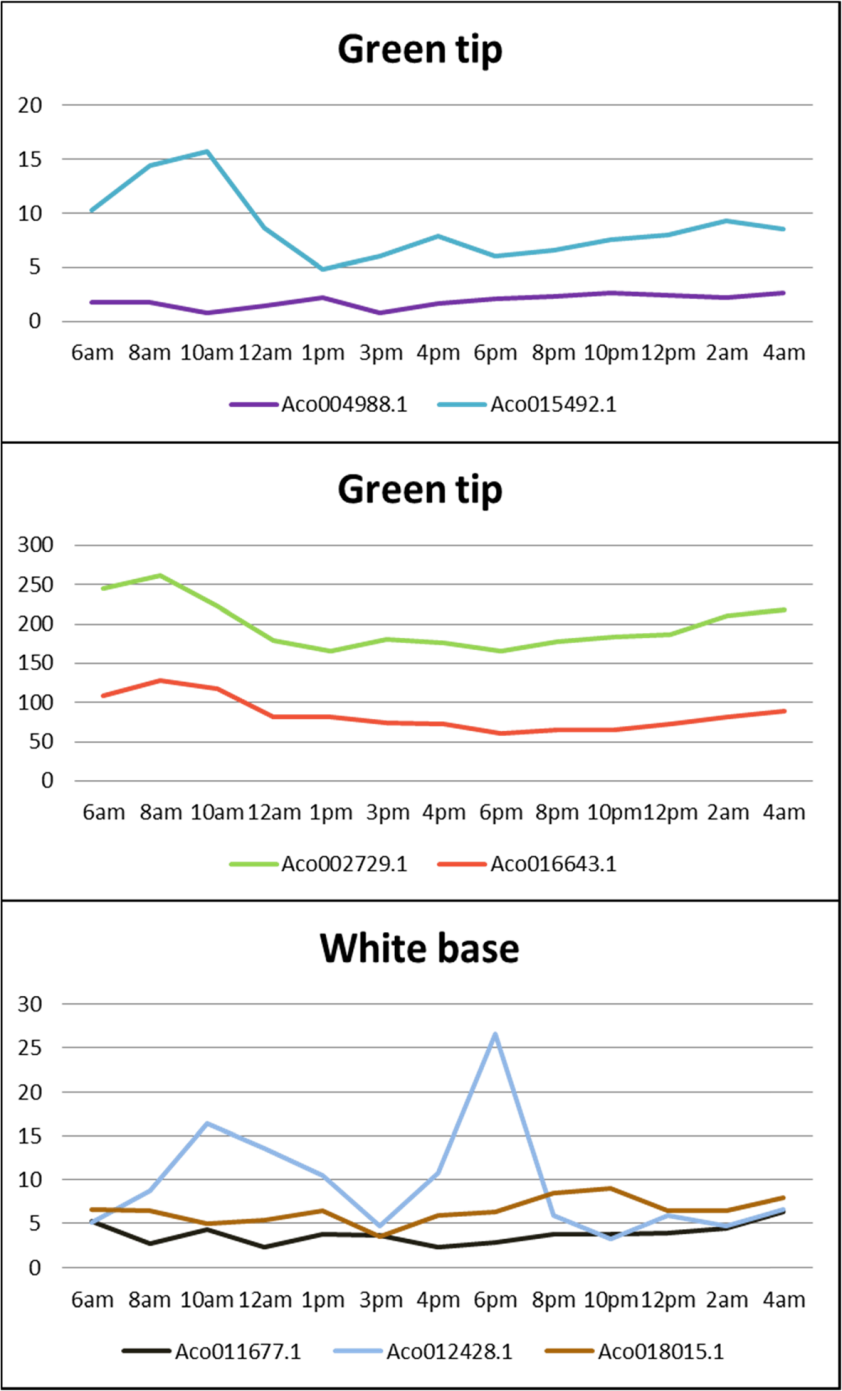
Diurnal expression patterns of MADS-box genes cycling in green tip or white base only

**Fig. 10:**
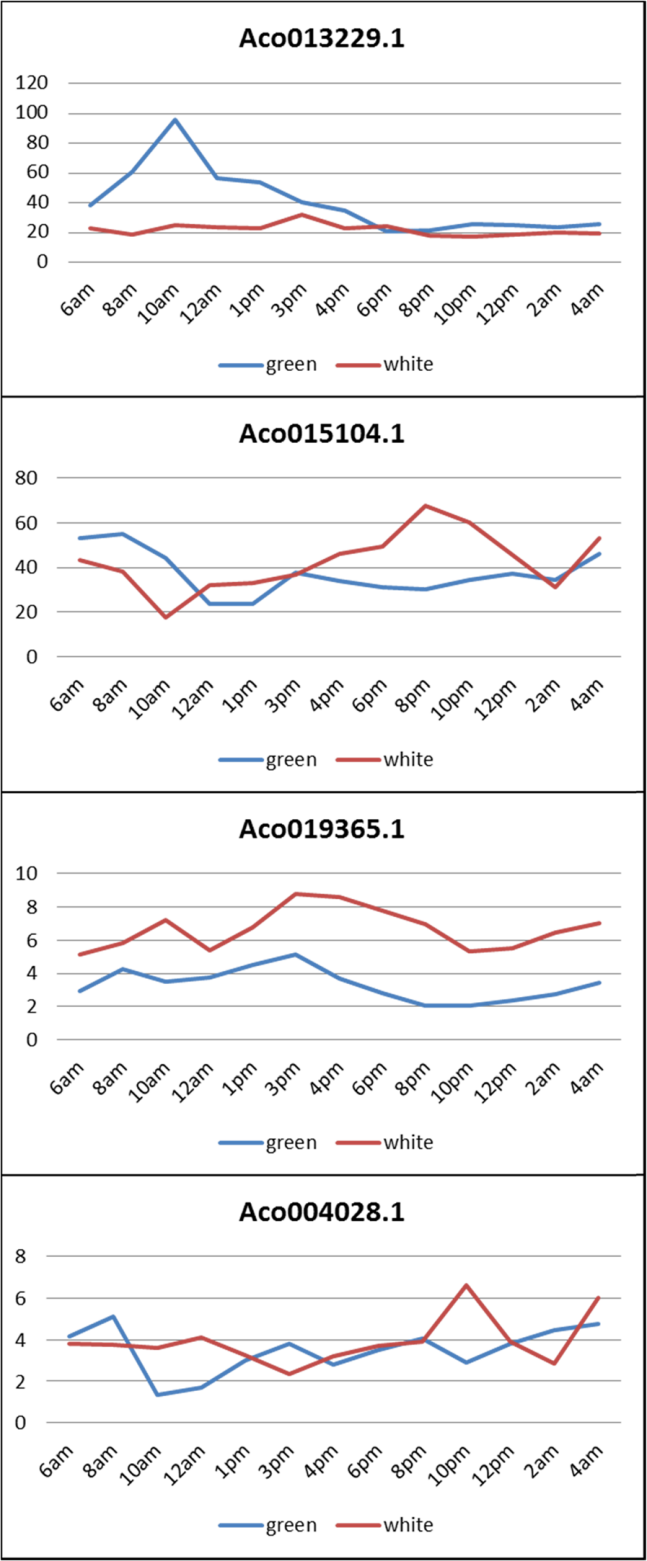
Diurnal expression patterns of MADS-box genes cycling in both green tip and white base leaf

Four genes were cycling in green tip leaf only, as shown in Fig. 9. *Aco015492.1* exhibited peak expression at 10 am and lowest expression at 1 pm, while *Aco004988.1* had lowest expression at 10 am and highest expression at 1 pm. *Aco002729.1* and *Aco016643.1* showed similar diurnal rhythm: peak expression at 8 am and lowest expression at 6 pm. There were three genes cycling only in white base leaf tissues (Fig. 9). What’s interesting is that *Aco012428.1* exhibited two peak expressions at 6 am and 10 am. Four genes were cycling in both green tip and white base leaves (Fig. 10). *Aco013229.1* had much higher expression in green tip than in white base during daytime from 6 am to 6 pm and similar expression level in both tissues at night. *Aco019365.1* exhibited similar expression pattern in both green tip and white base: highest expression at 3 pm, lowest expression at 10 pm, while *Aco004028.1* showed opposite expression profiles: highest expression in white base at 10 pm and in green tip at 8 am.

## Discussion

Compared with other plant species, pineapple has a relatively lower number of MADS-box genes. A total of 48 MADS-box genes were identified in pineapple, while 106, 75, 105 and 147 genes were discovered in *Arabidopsis*, rice, poplar and apple, respectively [28–31]. Previous studies showed that MADS-box family genes expand by whole genome duplication and gene duplication events [32, 33]. The difference of MADS-box gene numbers among species might be the result of recent duplications. Pineapple has undergone two ancient whole genome duplications (σ and τ), while rice has undergone a recent whole genome duplication (ρ) after the σ [24, 34]. It explains that the number of the pineapple MADS-box genes are less than that of rice and other species.

MADS-box genes are divided into two classes: type I and type II, and these two types have distinct evolutionary histories [10]. Type II MADS-box genes are mainly the result of whole genome duplications, while type I genes are caused by smaller scale and more recent duplications. It has been relatively easy to identify the orthologs of *Arabidopsis* type II MADS-box genes in different species, but orthologs of *Arabidopsis* type I MADS-box genes are difficult to be discovered in other species, mainly because that most duplicated type I genes are caused by genus-specific localized duplications [29]. The chromosomal distribution of MADS-box genes could also explain the idea that type I genes have resulted from smaller scale duplication. In *Arabidopsis*, type II genes were distributed across all chromosomes, whereas type I genes were clustered into only chromosomes I and V [12]. Type II MADS-box genes in pineapple were located on 18 chromosomes, while type I MADS-box genes were only scattered to 9 chromosomes.

Based on phylogenetic analysis, type II MADS-box genes in pineapple contain 32 MIKC-type and 2 Mδ-type and 32 MIKC-type genes could be divided into 11 subfamilies. There was no pineapple gene identified as *FLC* (*FLOWERING LOCUS C*) subfamily. *FLC* plays the important role in floral transition and serves as a major floral repressor in the autonomous and vernalization pathways [35]. The absence of pineapple *FLC* members indicated that pineapple could not require vernalization for flowering, a loss will not have any impact on this tropical plant. *SOC1* is a MADS-box gene in *Arabidopsis* regulated by two flowering regulators, *CONSTANS* (*CO*) and *FLC*, serving as floral activator and repressor, respectively [36, 37]. Three *SOC1*-like pineapple genes were identified, while no *CO* member was found in pineapple. The regulatory mechanisms of flowering in pineapple might be different from that of *Arabidopsis*. Type I MADS-box genes could be divided into Mα, Mβ and Mγ. In *Arabidopsis*, type I genes play important role in plant reproduction as well as the maintenance of species barriers and are required for endosperm development [38–40]. Studies showed that type I MADS-box genes had faster birth and death compared with type II genes, which could further explain the different pattern of type I and type II genes in phylogenetic tree.

Knowing where the genes are expressed is important for understanding the molecular mechanisms of biological development. The expression patterns of MADS-box genes in different organs of pineapple indicated that the pineapple MADS-box genes were expressed differently in the different tissues. The higher expression level of MADS-box genes in the flowers indicated that MADS-box genes play the important roles in the flower development in pineapple. The MADS-box transcription factors were regarded as the genes involved in floral organ identity. For example, AGAMOUS 1 (TAG1) was involved in flower and fruit development of tomato [41]. Although most MADS-box genes were reported to be related to flower development, we want to know whether some MADS-box genes were also involved in the CAM photosynthesis. Thus, the expression patterns of MADS-box genes in both photosynthetic and non-photosynthetic leaf tissues was studied to investigate the potential roles of MADS-box genes in pineapple CAM photosynthesis. The results showed that many MADS-box genes have different expression levels in white base (non-photosynthetic) and green tip (photosynthetic) leaf tissues and more genes expressed higher in white base than in green tip, which indicating that MADS-box genes are not typical genes involved in photosynthesis, but some genes might play certain roles in pineapple CAM photosynthesis.

Circadian clock, as an important regulator, plays a crucial role in the biological mechanisms such as developmental or metabolic process [27]. 23% (11 out of 48) of MADS-box genes displayed diurnal expression, the proportion of pineapple MADS-box genes with circadian rhythm was lower than expected partially because only leaf samples were used for diurnal expression analysis. However, the results still indicated that some MADS-box genes in pineapple could be involved in the circadian clock. In *Arabidopsis*, circadian clock has been widely studied and mutants with perturbed circadian rhythms were large-scale screened [42].

## Conclusions

In this study, we conducted the whole-genome analysis of MADS-box genes and then identify 48 MADS-box genes in the pineapple genome. Forty-eight MADS-box genes can be divided into 14 type I and 34 type II MADS-box genes. a majority of pineapple MADS-box genes are highly expressed in flowers, which indicates that pineapple MADS-box genes are closely related to flowering development. Some MADS-box genes express differently in photosynthetic and non-photosynthetic leaf tissues, suggesting that MADS-box genes could be involved in CAM photosynthesis. 23% of pineapple MADS-box genes are regulated by the circadian clock. These findings will facilitate research on the development of unusual spiral inflorescences on pineapple fruit and CAM photosynthesis.

## Methods

### Whole-genome identification of MADS-box genes in pineapple

The protein sequences of pineapple, rice and Arabidopsis were obtained from Phytozome (https://phytozome.jgi.doe.gov/pz/portal.html), RGAP (http://rice.plantbiology.msu.edu/) and TAIR (http://www.arabidopsis.org/) databases, respectively. To identify the MADS-box genes in pineapple, the Hidden Markov Model (HMM) profiles of the SFR (type I) domain (PF00319) and the MEF2 (type II) domain (PF09047), downloaded from Pfam database (http://pfam.xfam.org, Pfam 31.0), were used to search the pineapple genome database [43, 44]. All of the proteins with an E-value lower than 0.01 were selected. Secondly, using all Arabidopsis and rice MADS-box genes as queries, the predicted pineapple MADS genes were checked by BLASTP searches (https://blast.ncbi.nlm.nih.gov/Blast.cgi). Finally, the predicted MADS models detected were examined manually. The retrieved pineapple MADS genes were further verified by the NCBI Conserved Domain Database (https://www.ncbi.nlm.nih.gov/cdd).

### Classification of pineapple MADS-box genes

MADS-box genes in *Arabidopsis* and rice were used for classifying the pineapple MADS-box genes. Multiple sequence alignments were performed based on protein sequences of MADS-box genes in pineapple, *Arabidopsis* and rice using MAFFT (https://www.ebi.ac.uk/Tools/msa/mafft/). A phylogenetic tree was then constructed based on multiple sequence alignments using RAxML with the parameters: pair wise gap deletion and 1000 bootstrap iterations [45]. The phylogenetic tree was further annotated by iTOL program (http://itol.embl.de/).

### Gene structure and conserved motif analysis

To identify the gene structure of pineapple MADS-box genes, the full-length coding sequence (CDS) and genomic sequence of MADS-box genes were used to perform gene structure analysis by Gene Structure Display Server program (http://gsds.cbi.pku.edu.cn/) [46]. Online software MEME was used to search motifs in pineapple MADS-box genes (http://meme-suite.org/tools/meme) with the parameters: maximum number of motifs – 20 and optimum motif width set at ≥6 and ≤ 200. The motifs of MADS-box genes were annotated by the SMART program (http://smart.embl-heidelberg.de/).

### Location of pineapple MADS-box genes on chromosomes

The pineapple genome has been mapped to 25 chromosomes [24]. To explore the chromosomal location of MADS-box genes, online software MA2C (MapGene2Chromosome v2) (http://mg2c.iask.in/mg2c_v2.0/) was used to map pineapple MADS-box genes onto chromosomes.

### Expression analysis of pineapple MADS-box genes in four tissues

Expression patterns of MADS-box genes at different tissues (flower, root, leaf and fruit) were analyzed using RNA-Seq data obtained from Ming et al. [24]. Flower, root and leaf tissues were collected from cultivar F153 and fruit tissue was obtained from cultivar MD-2. The tissues were stored at -80 °C for RNA extraction and transcriptome analysis. The FPKM values were calculated by the Cufflinks/Cuffnorm pipeline (http://cufflinks.cbcb.umd.edu/). Genes with no expression (FPKM values equal “0” in all tissues) were filtered. The expression pattern of pineapple MADS-box genes in different tissues was visualized by a heat map.

### Diurnal expression analysis of MADS-box genes

Green tip (photosynthesis) and white base (non- photosynthesis) leaf tissues were collected from field pineapple cultivar MD-2 grown in Hawaii over a 24-h period to examine the diurnal expression patterns of pineapple genes. Five individual plants were collected as one replicate, and three biological replicates were collected. The method of analyzing circadian rhythm was adopted from Sharma et al. [27]. Online software Haystack was used to analyze the time series expression data (http://haystack.mocklerlab.org/), with parameters: correlation cut off 0.7, *P* value cut off 0.05, fold change cutoff 2 and background cutoff 1.

### Plant material, RNA extraction and quantitative RT-PCR analysis

The flower and leaves of pineapple cultivar MD-2 were obtained from the greenhouse of Fujian Agriculture and forestry University (26°4′54″N, 119°13′47″E) on October 25th, 2019. The average temperature of greenhouse is around 28 °C, and the light cycle is from 4:00–20:00. The ways of collecting pineapple samples and designing biological replicates was the same as the protocols in the paper of Ming et al. [24].

Total RNA was extracted using Trizol protocol. Reverse transcription was performed from 2μg of RNA using TransScript One-Step Supermix kit. The cDNA was diluted ten-fold for the following qRT-PCR verification. Primers for pineapple MADS-box genes were designed using online website (https://www.idtdna.com/PrimerQuest/Home/Index). Primers information are listed in the Additional file 1: Table S1. The qRT-PCR reaction was performed in the 20 μL volume containing 1 μL of cDNA, 1 μL of each primezr and 10 μL of SYBR Green mix and was under the following program: 95 °C for 3 min; 32 cycles at 95 °C for 15 s, 60 °C for 15 s, and 72 °C for 30 s; 72 °C for 10 min.

The expression of MADS-box genes in different tissues (flower and leaves), green tip and white base leaves at different time points (6 am, 12 am, 6 pm, 12 pm) were verified by qRT-PCR. All the reactions were performed in three biological replicates.

## Supplementary information

**Additional file 1:** **Table S1.** The primer sequences for qRT-PCR

## Data Availability

The datasets analyzed in this study are publicly available in NCBI under BioProject PRJNA305042.

## Abbreviations

AGL11: Agamous like-11
AGL12: Agamous like-12
ANR1: Arabidopsis Nitrate Responsive1
CAM: Crassulacean Acid Metabolism
CO: CONSTANS
FLC: Flowering Locus C
HMM: Hidden Markov Model
MEME: Multiple Em for Motif Elicitation
NCBI: National Center for Biotechnology Information
SEP: SEPALATA
SMART: Simple Modular Architecture Research Tool
SOC1: Suppressor of Overexpression of Co1
SVP: Short Vegetative Phase
TT16: Transparent Testa16
